# Millet-based supplement restored gut microbial diversity of acute malnourished pigs

**DOI:** 10.1371/journal.pone.0250423

**Published:** 2021-04-29

**Authors:** Xuejing Li, Yan Hui, Bingfeng Leng, Junli Ren, Yanni Song, Lianqiang Che, Xi Peng, Baojia Huang, Songling Liu, Lin Li, Dennis Sandris Nielsen, Yong Li, Xiaoshuang Dai, Shancen Zhao

**Affiliations:** 1 BGI Institute of Applied Agriculture, BGI-Shenzhen, Shenzhen, China; 2 Department of Food Science, University of Copenhagen, Copenhagen, Denmark; 3 Neomics Institute, Life and Science Park 301, Pingshan, Shenzhen, China; 4 BGI Education Center, University of Chinese Academy of Sciences, Shenzhen, China; 5 Animal Nutrition Institute, Sichuan Agricultural University, Ya’an, Sichuan, China; 6 College of Life Science, China West Normal University, Nanchong, China; 7 ShenZhen Engineering Laboratory for Genomics-Assisted Animal Breeding, BGI-Shenzhen, Shenzhen, China; USDA-Agricultural Research Service, UNITED STATES

## Abstract

The tight association between malnutrition and gut microbiota (GM) dysbiosis enables microbiota-targeting intervention to be a promising strategy. Thus, we used a malnourished pig model to investigate the host response and GM alterations under different diet supplementation strategies. Pigs at age of 4 weeks were fed with pure maize diet to induce malnutrition symptoms, and followed by continuous feeding with maize (Maize, *n* = 8) or re-feeding using either corn-soy-blend (CSB+, *n* = 10) or millet-soy-blend based (MSB+, *n* = 10) supplementary food for 3 weeks. Meanwhile, 8 pigs were fed on a standard formulated ration as control (Ref). The effect of nutritional supplementation was assessed by the growth status, blood chemistry, gastrointestinal pathology, mucosal microbiota composition and colon production of short-chain fatty acids. Compared with purely maize-fed pigs, both CSB+ and MSB+ elevated the concentrations of total protein and globulin in blood. These pigs still showed most malnutrition symptoms after the food intervention period. MSB+ had superior influence on the GM development, exhibiting better performance in both structural and functional aspects. MSB+ pigs were colonized by less *Proteobacteria* but more *Bacteroidetes*, *Firmicutes* and *Lachnospira* spp. Pearson’s correlation analysis indicated a strong correlation between the abundance of mucosal e.g., *Faecalibacterium* and *Lachnospira* spp. and body weight, crown-rump length and total serum protein. In conclusion, the malnutrition symptoms were accompanied by an aberrant GM, and millet-based nutritional supplementation showed promising potentials to restore the reduced GM diversity implicated in pig malnutrition.

## Introduction

Malnutrition accounts for nearly half of the deaths among children under 5-year-old in low- and middle-income countries [1]. In 2017, 7.5% of children under the age of 5 were affected by wasting syndrome, with the regional prevalence ranging from 1.3% in Latin America to 9.7% in Asia [2]. More than 90% of global children stunting occurred in Africa and Asia [2]. Malnutrition usually develops from inadequate protein and micronutrients intake due to unavailability of food [3, 4]. Epigenetic modification during the pre- and neo-natal period, such as metabolic imprinting, will also increase the risk of malnutrition and diet-related non-communicable diseases [5, 6].

Corn soy blend with extra micronutrients (CSB) is a widely-applied ready-to-use supplementary food (RUSF) to treat malnourished children. But the treated children still have anemia or concurrency of malnutrition [7, 8]. In recent years, increasing evidence has indicated an essential role of the gut microbiota in the development of malnutrition. The gut microbiota works as a virtual organ involved in the regulation of several host parameters, including polysaccharide digestion [9], immune system development [10], defense against infections [11], synthesis of vitamins and fat storage [11, 12]. Blanton et al. has emphasized clearly that a causal relationship exists between the gut microbiota immaturity and malnutrition. Two kinds of invasive species, *Ruminococcus gnavus* and *Clostridium symbiosum* transferred from malnourished donors, were found to ameliorate growth and metabolic abnormalities in recipient animals [13].

Millet is one of the most important drought-resistant grains in arid and semiarid areas of Asia and Africa. It has attracted specific attention given the balanced nutritional value and potential health benefits such as anti-oxidant and anti-arteriosclerotic possibility [14]. Several studies have shown that millet, as a kind of functional food, contributed to maintaining homeostasis of blood glucose [15, 16], delaying gastric emptying [17, 18], and enhancing immune competence [18]. Thus, we hypothesized that millet supplementation in feed cloud benefit the host digestive and immune system, sustain the GM development and relieve the malnutrition symptoms. In order to assess the effect of millet-based feed, we included pure maize malnutrition diet, standard pig formulated ration and corn-soy-blend feed as comparison. The host response and colon mucosal microbiota changes of different feeding strategy were compared after a three-week intervention period.

## Materials and methods

### Animals experimental design and procedures

**Fig 1:**
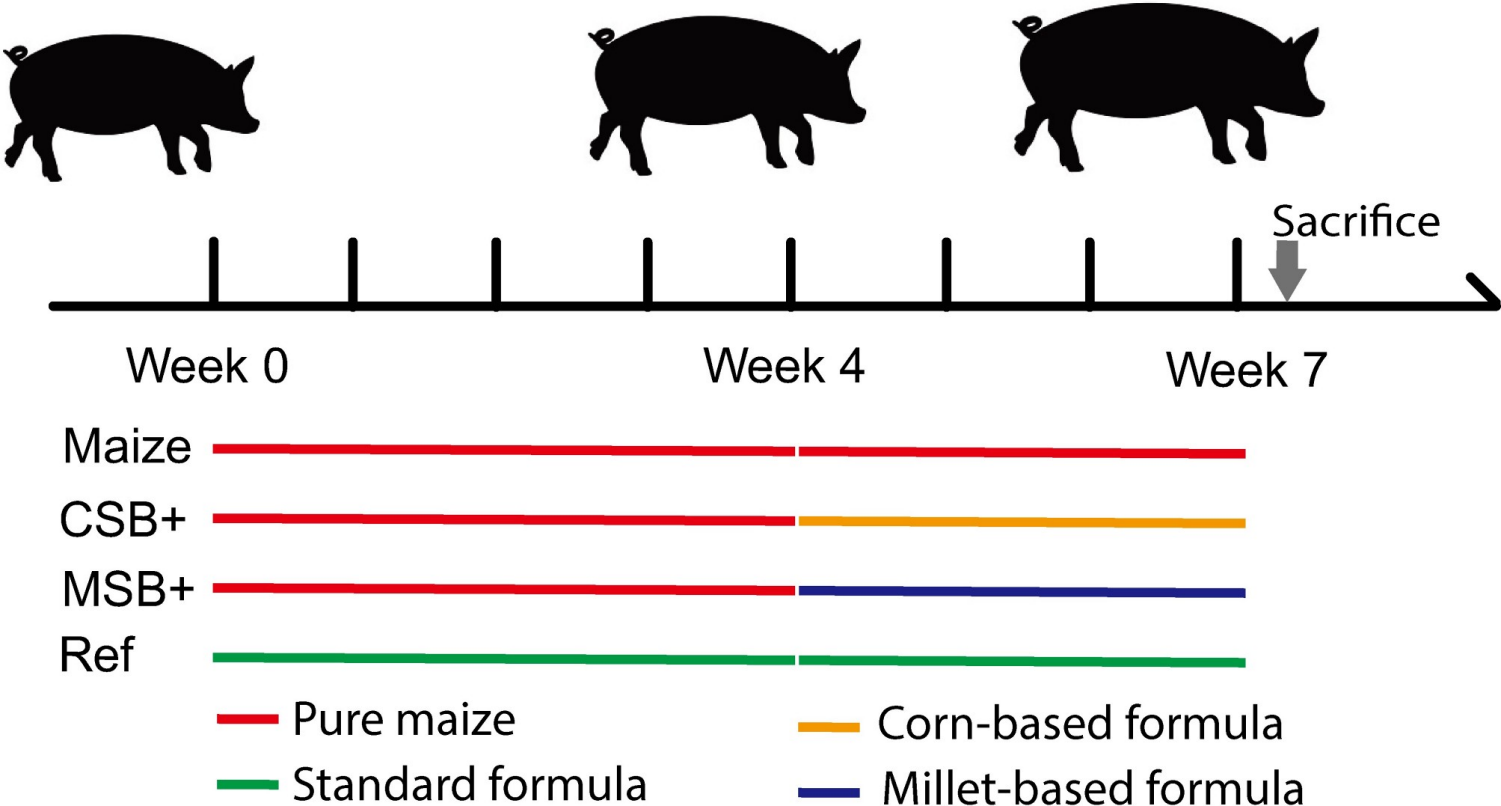
Different treatments between malnourished pigs and controls.

The whole animal experiment was permitted and inspected by the BGI bioethics and biosafety board (No. FT18099). Thirty-six female Durox × Danish Landrace × Yorkshire crossbred pigs (Shenzhen Agriculture and Animal Husbandry Co., Ltd., China), weaned at the age of 4 weeks with body weight 6.5 ± 0.2kg (Mean ± SD), and fed *ad libitum* with adequate formula for one-week acclimatization. Then these pigs were stratified by body weight and were randomly allocated to be fed by the pure maize diet (*n* = 28) or the previous formulated ration (Ref, *n* = 8). After 4 weeks of respective feeding, the malnourished pigs were randomized into 3 groups based on similar body weights at 5.5 ± 1.7kg (Mean ± SD). In the following 3 weeks, malnourished pigs received CSB+ (corn soy blend with extra micronutrients, CSB+, *n* = 10), MSB+ (millet soy blend with extra micronutrients, MSB+, *n* = 10), maize (Maize, *n* = 8) diet accordingly while Ref continued with the optimal formulated diet as positive control. Detailed study design is presented in Fig 1. All the groups had *ad libitum* access to feed and water during the study period. The nutritional composition of diets is presented in Table 1 and the average feed consumption was calculated by pen (S1 Fig). After 3 weeks of refeeding, all the pigs were euthanized for sample collection after deep anesthesia with lethal dose of intracardiac barbiturate. CSB+ was produced according to the diet composition provided by the United States Agency for International Development (USAID), and MSB+ showed slight difference in components given the natural difference of millet. No fortifiers were added according to requirements of the Chinese standards for the Use of Food Nutrition Fortifiers (GB 14880).

**Table 1:** Diet compositions used in our treatments.

Component	Diets			
	CSB+	MSB+	Maize	Ref
Energy (kJ)	1678	1649	1480	1540
Protein (g)	13.2	13.3	8	20.4
Fat (g)	5.33	6.5	3.3	5.6
Carbohydrate (g)	74	70	75.6	57.9
Sodium (mg)	5.89	6	10	456
Vitamin A (μg)	1098.54	1108.45	n.d.	316
Vitamin D (μg)	11	1.5	n.d.	4.94
Vitamin E (mg)	8.56	8.44	n.d.	12.1
Vitamin K (μg)	39.4	7.64	n.d.	n.d.
Vitamin B1 (mg)	0.48	0.525	n.d.	1.3
Vitamin B2 (mg)	n.d.	0.48	n.d.	n.d.
Vitamin B12 (μg)	2	2	n.d.	12
Vitamin B6 (mg)	n.d.	0.5	n.d.	n.d.
Nicotinic acid (mg)	9.11	9.11	n.d.	9.1
Folic acid (μg)	208.54	205.22	n.d.	438
Pantothenic acid (mg)	1.95	2.01	n.d.	5.59
Biotin (μg)	8.2	3.07	n.d.	46.3
Ca (mg)	509.75	508.75	43	846
P (mg)	508.49	508.49	330	816
K (mg)	610.83	610.83	352	1030
Mg (mg)	81.11	120.28	115	156
Fe (mg)	10.5	10.7	2.96	38.8
Zn (mg)	6.5	6.6	n.d.	154
I (μg)	40	n.d.	n.d.	87

Per 100g edible portion. (n.d., not determined)

### Growth and malnutrition estimation

Growth statistics, including body weight and crown-rump length (CRL), were measured weekly. These anthropometric measurements were used to assess the degree of malnutrition as described in details below. Based on the lengths of the malnourished pigs, theoretical weights were calculated:

$$Theoretical\;weight=e^{3.77}\times {CRL}^{2.401}$$

where respective unit for theoretic weight and CRL is kg and m.

The estimation of the degree of wasting was calculated:

Wastingdegree=Observedweight÷Theoreticalweight×100%.

The degrees of underweight and stunting were calculated as percentages of the mean values of the age-matched reference pigs:

Underweightdegree=Weightofundernourishedpigs÷Meanweightofthereferencepigs×100%;

Stuntingdegree=Lengthofundernourishedpigs÷Meanlengthofthereferencepigs×100%

[19].

### Blood sampling, tissue collection and histology assessment

For biochemical and systemic immune analysis, blood was collected by venipuncture of jugular venous from anaesthetized pigs just prior to euthanasia. For each sample, blood was collected by a 5-mL coagulation and a 5-mL heparin sodium tube respectively. Coagulation tubes were centrifuged at 2154 g for 15 min at 4 °C and supernatant (serum) was harvested and stored at -80 °C for blood biochemistry analysis. Similarly, heparin sodium tubes were centrifuged at 1600 g for 10 min at 4 °C and supernatant (plasma) was harvested for systemic immune analysis.

The heart, liver, kidneys, spleen and lungs were removed from the pigs and weighed immediately after euthanasia. Distal colons were fixed separately in 5% paraformaldehyde, and embedded with paraffin wax for histology assessment. The histological analyses were blinded and conducted by experienced personnel in one commercial company (Chengdu Yonkers Bio-Technology Co., LTD). In short, the paraffin-embedded tissues were cut on a microtome (Leica RM2235, Germany) into 5 μm sections and stained with hematoxylin and eosin. The whole tissue film was observed under the light microscope (Olympus CX22, Japan) and the histopathological changes were photographed by the microscopic imaging system (Leica DM1000, Germany). After the distal colon sections were stained with Alcian blue and Periodic acid-Shiff (AB-PAS), the ability of goblet cells to secrete mucus was evaluated by measuring the area of acidic mucin, neutral mucin and mucous layer and quantifying the relative areas of acidic mucin and neutral mucin to the total area of mucous layer. The colonic content and mucosa collection followed one published method [20] with minor revision. In short, the middle part of the colon was resected and the colon luminal content contents were collected for short-chain fatty acid determination (SCFA) by the incision. Then the colon segment was opened to remove residual contents and the mucosal scrapings were collected using one glass slide.

### Blood biochemistry and systemic cytokines determination

For blood biochemistry analysis, the concentrations of albumin, total protein, urea, glucose, triglyceride, total cholesterol, low-density protein cholesterol, creatinine, C-reactive protein and total bile acid (all purchased from Maccura, Sichuan, except total bile acid from KHB, Shanghai) in serum were measured using an Automatic Analyzer 3100 (HITACHI, Japan). The concentrations of LPS, leptin (KENUODIBIO, SU-B50226; SU-B50089) and inflammatory factors (IL-1β, TNF-α, and IL-6) were determined in a single assay using ELISA kits (AMEKO, AE90731Po; AE90301Po; AE90247Po) according to the manufacture’s instruction.

### Short-chain fatty acid determination by gas chromatography-mass spectrometry

The colon luminal contents were prepared for GC-MS analyses as previously described [21] with slight modification. For colon contents, the extraction procedures were performed at 4 °C to protect the volatile SCFAs. In brief, 200 mg samples were mixed with 2 mL of 5μg/ml ethyl acetate containing 2-Ethylbutyric acid as an internal standard. 200 μL of 1 M sodium chloride solution saturated with hydrochloric acid was added then. The mixed solution was sonicated for 1h and then centrifuged for 10 min at 10,000 g at 4 °C. MgSO_4_ was added to supernatant (20 times dilution) and then centrifuged (18000 g,10 min, 4 °C). Collected supernatant 72 μL and 18 μL of MTBSTFA (Aladdin, Shanghai, China) was added. The solution was heated for 20 min at 80 °C for derivatization. Then, the samples were cooled and transferred for GC-MS analyses.

GC-MS analyses were performed as previously described [22] with several modifications. The SCFA analysis was carried out using a TSQ 9000 GC-MS/MS. A nonpolar DB-5MS capillary column (30 m × 0.25 mm × 0.25 μm, J&W Scientific, Folsom, CA, USA) was used for chromatographic separation. Helium (1.0 ml/min) was used as the carrier gas. The chromatographic stepwise thermal conditions were as follows: 60 °C for 2 min, 6 °C/min until 90°C for 2 min, 6 °C/min until 120 °C and 50 °C/min until 280 °C for 3 min. The mass spectrometer was set to scan mode from m/z 30–300 and in selected ion monitoring mode at m/z of 131 for propionic acid, m/z of 145 for butyric acid and isobutyric acid, m/z of 159 for isovaleric acid and valeric acid, and m/z of 173 for 2-Ethylbutyric acid (internal standard). The concentration of acetic acid was not reported due to the standard curve lacking adequate linearity.

### 16S rRNA mucosal microbiome sequencing

Total cellular DNA was extracted from the mucosal scrapings with the E.Z.N.A. Stool DNA Kit (Omega) according to the company instruction. The bacterial hypervariable V3-V4 region of 16s rRNA was chosen for paired-end 300bp amplicon sequencing on MiSeq platform (Illumina, CA USA) using the primers: 341_F: 5’- CCTACGGGNGGCWGCAG-3’ and 802_R: 5’- TACNVGGGTATCTAATCC-3’. The library preparation followed the method published previously [23].

### Statistics

All continuous data except microbiome data was analyzed in R (version 3.6.0) with respective packages. The body and organ weight, biochemical, systemic immune and SCFA data was analyzed by ANOVA and multiple pairwise t-tests were adjusted by Tukey procedure using the “compareGroups” and “createTable” command in compareGroups [24] (R package, version 4.0). The intestine inflammation indexes were analyzed by Wilcoxon test for pairwise tests and the *p* values were adjusted by Benjamini-Hochberg procedure for each independent experiment, where R package rstatix [25] (version 0.1.0) was implement to complete analysis. Zeros in SCFAs data were replaced by the lowest value in all the samples. Data were shown as mean ± SD and mean ± SEM accordingly. Adjusted *p* values below 0.05 were regarded as statistically different.

For microbiome analysis, the raw sequencing reads were merged and trimmed, followed by removing chimera and constructing zero-radius Operational Taxonomic Units (zOTUs) with UNOISE [26] implemented in Vsearch [27] (v2.6.0). The Green Genes database (13.8) 16S rRNA gene database was used as reference for annotation. QIIME 2 [28] (2018.11) combined with R packages (ggplot2 [29], vegan [30]) was used for analysis. Rare zOTUs with frequency below 0.1% of the minimal sample depth were removed and filtered zOTU table was rarified to adequate sample depth (47000 count) for alpha and beta diversity calculation. Principal coordinate analysis (PCoA) was conducted on unweighted and weighted UniFrac distance and PERMANOVA was performed to determine the dissimilarity between groups and *p* values were adjusted after pairwise tests. Differentially abundant taxa were identified by ANCOM using default setting in QIIME 2 [28]. For the taxa found by ANCOM, Wilcoxon tests were conducted for pairwise tests and the *p* values were adjusted with Benjamini-Hochberg procedure. The rarified zOTU table was used to predict the functional genetic make-up using PICRUSt 2 [31]. The KEGG Orthology (KO) table was downloaded from KEGG website (March, 2019) for annotation. Only the predicted KOs annotated in the KO table were kept to calculate the pathway abundance. The differentially abundant pathways were identified by LEfSe [32] with a strict threshold of linear discriminant analysis (LDA) score above 3.

Further, the rarefied zOTU table was collapsed at the genera level and rare genera were removed according to a pre-set cut-off (mean relative abundance > 0.1% and percentage of presence > 30%). Zeros were regarded as NA. The Pearson’s correlation analysis implemented in Rhea [33] was conducted between relative abundance of genera and the phenotype data after centered log-ratio data transformation. Correlation matrix was visualized with R package corrplot [34] and the correlation pairs with *p* value < 0.05 and absolute value of coefficient > 0.5 were plotted in scatter plots by Rhea [33].

## Results

### Phenotypic data analysis and SCFA quantification with GC-MS

After 3-week nutritional supplementation, CSB+, MSB+ and Maize fed pigs, when compared with Ref, still remained low level of CRL and body and organ weight (*p* < 0.01, S1 Table). Under *ad libitum* feeding, Ref pigs showed faster feed consumption relative to other groups (S1 Fig). The malnutrition index didn’t show distinct difference between the malnutrition pig groups except for a lower weight-for-age index of MSB+ relative to Maize (S2 Fig). Serum biochemical analysis showed significant differences between the treatment groups except for triglyceride, urea and total bile acid (*p* < 0.05, S1 Table). Compared with Maize, both CSB+ and MSB+ improved total serum protein and globulin (*p* < 0.05, Table 2) with globulin concentrations being similar to Ref (*p* = 0.70 and 0.51 respectively). The systemic concentrations of IL-1beta and IL-6 were up-regulated in malnourished pigs relative to Ref (S1 Table). Refeeding with MSB+ and CSB+ only led to numerical reduction of the proinflammatory IL-1beta and IL-6 in blood (Table 2). Short-chain fatty acids in the colon of malnourished pigs were quantified by GC-MS (Table 3). Nutritional supplementation increased the abundance of propionic acid, which was significantly up-regulated in the MSB+ group relative to Maize. For other SCFAs, neither CSB+ nor MSB+ showed significant differences relative to Maize.

**Table 2:** Pig growth, blood biochemical and systemic immune index.

	CSB+ N = 10	Maize N = 8	MSB+ N = 10	Ref N = 8	p.CSB+ vs Maize	p.MSB+ vs Maize
Body weight (kg)	6.00 ± 1.51	7.20 ± 1.08	5.63 ± 1.07	28.8 ± 3.82	0.63	0.41
CRL (cm)	49.4 ± 3.60	53.1 ± 3.73	51.6 ± 4.86	71.2 ± 1.75	0.19	0.86
Heart weight (kg)	0.04±0.01	0.04±0.01	0.03±0.01	0.13±0.02	0.89	0.52
Liver weight (kg)	0.17±0.05	0.18±0.05	0.20±0.04	0.74±0.12	0.97	0.94
Spleen weight (kg)	0.02±0.00	0.02±0.01	0.02±0.00	0.14±0.22	1.00	1.00
Lung weight (kg)	0.12±0.04	0.11±0.02	0.11±0.02	0.34±0.03	0.89	1.00
Kidney weight (kg)	0.04±0.01	0.04±0.01	0.03±0.01	0.13±0.01	0.93	0.39
Total protein (g/L)	53.1 ± 4.75	44.6 ± 5.05	53.1 ± 5.50	69.7 ± 5.87	0.01	0.01
Albumin (g/L)	14.4 ± 1.90	11.9 ± 1.82	15.0 ± 1.74	28.9 ± 4.02	0.18	0.06
Globulin (g/L)	38.7 ± 3.47	32.7 ± 3.44	38.1 ± 4.76	40.8 ± 4.32	0.02	0.04
Albumin/Globulin	0.37 ± 0.04	0.37 ± 0.03	0.40 ± 0.06	0.72 ± 0.12	1.00	0.77
Glucose (mmol/L)	5.21 ± 1.84	5.99 ± 0.79	7.11 ± 1.71	6.58 ± 0.97	0.68	0.38
Urea (mmol/L)	6.89 ± 2.53	5.47 ± 1.90	6.55 ± 1.00	4.77 ± 0.60	0.32	0.56
Creatinine (mmol/L)	50.4 ± 16.5	60.7 ± 15.6	50.7 ± 12.4	93.6 ± 5.70	0.39	0.42
Cholesterol (mmol/L)	2.40 ± 0.46	2.37 ± 0.31	2.69 ± 0.36	3.02 ± 0.79	1.00	0.55
Triglyceride (mmol/L)	0.61 ± 0.34	0.48 ± 0.15	0.53 ± 0.15	0.66 ± 0.31	0.70	0.98
Low-density protein (mmol/L)	0.98 ± 0.20	1.06 ± 0.21	1.10 ± 0.27	1.54 ± 0.57	0.96	0.99
C-reaction protein (mmol/L)	8.42 ± 4.29	4.69 ± 2.82	3.73 ± 1.65	5.25 ± 3.31	0.08	0.92
Total bile acid (mmol/L)	24.6 ± 9.70	33.4 ± 24.3	44.3 ± 23.2	28.5 ± 11.2	0.74	0.60
IL-1beta (pg/mL)	66.9 ± 63.2	143 ± 84.1	87.1 ± 46.1	48.2 ± 58.8	0.07	0.26
IL-6 (pg/mL)	7.57 ± 7.71	10.8 ± 5.18	8.95 ± 5.67	1.78 ± 1.73	0.64	0.90
TNF-alpha (pg/mL)	19.2 ± 5.52	18.7 ± 11.0	22.4 ± 4.90	16.4 ± 8.37	1.00	0.74
Leptin (ng/mL)	22.2 ± 9.86	15.8 ± 9.95	20.8 ± 11.2	17.8 ± 8.58	0.54	0.71
Lipopolysaccharide (pg/mL)	1032 ± 587	669 ± 465	925 ± 626	722 ± 373	0.49	0.74

Data is shown as mean ± SD.

**Table 3:** SCFA concentrations in the colon content.

	CSB+ N = 10	Maize N = 8	MSB+ N = 10	p.CSB+ vs Maize	p.MSB+ vs Maize
Propionic acid (μg/mL)	105 ± 57.3	65.6 ± 29.0	129 ± 30.1	0.14	0.01
Butyric acid (μg/mL)	25.4 ± 13.3	31.6 ± 12.9	32.9 ± 9.84	0.54	0.97
Pentanoic acid (μg/mL)	18.3 ± 9.04	20.0 ± 8.67	24.2 ± 8.04	0.91	0.57
Isobutyric acid (μg/mL)	6.49 ± 2.57	10.3 ± 4.80	9.93 ± 3.94	0.12	0.98
Isopentanoic acid (μg/mL)	12.1 ± 3.52	18.0 ± 6.94	15.9 ± 3.54	0.05	0.63

Data is shown as mean ± SD.

**Fig 2:**
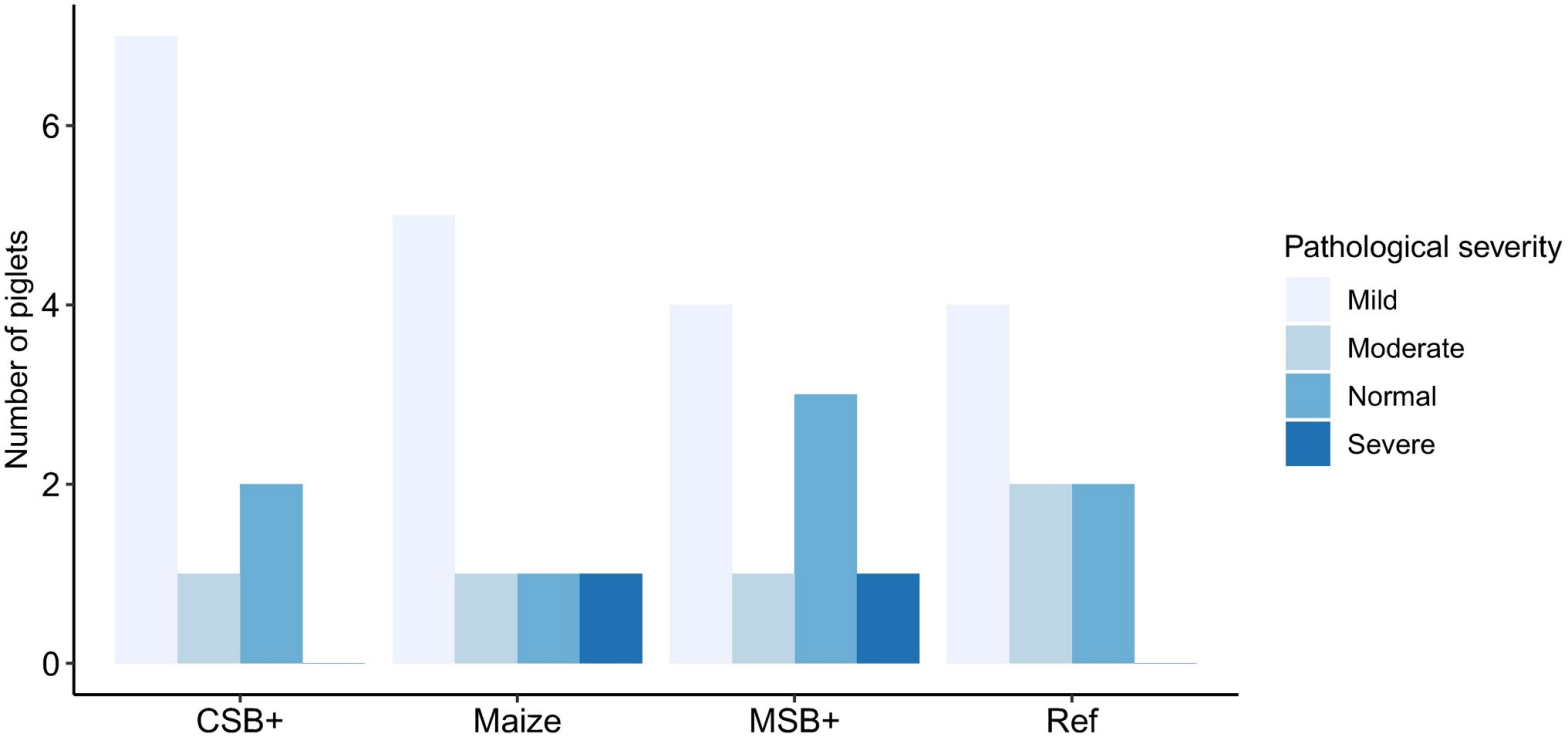
Pathological severity of distal colon in the experiment groups. One MSB+ sample was unavailable for histological assessment due to sample missing in the preservation.

The gastrointestinal pathological severity was evaluated according to the histological characteristics of epithelial cells, mucosal and submucosal structures in the colon. Mild, moderate and severe pathological change was defined as shown in S3 Fig. In the maize group, 7 of 8 pigs showed pathological changes in the colon, and of which 5 presents mild, 1 was moderate and 1 was severe. However, pathological changes in 6 of 8 pigs in Ref were also observed, 4 had mild pathological changes and the rest presented moderate. In the refeeding groups, pathological changes were found in 8 pigs out of 10 in CSB+ and 6 out of 9 in MSB+ group. Seven were defined as mild and 1 as moderate in CSB+ group while 4 were mild, 1 moderate and 1 severe in MSB+ group (Fig 2). Malnourished pigs had relatively thinner intestinal wall relative to Ref but only CSB+ showed significant difference from Ref (P<0.05, S4 Fig). The density of both neutral and acidic goblet cells showed no significant differences between groups (S5 Fig).

### Mucosal microbial community profiled by 16S rRNA gene amplicon sequencing

**Fig 3:**
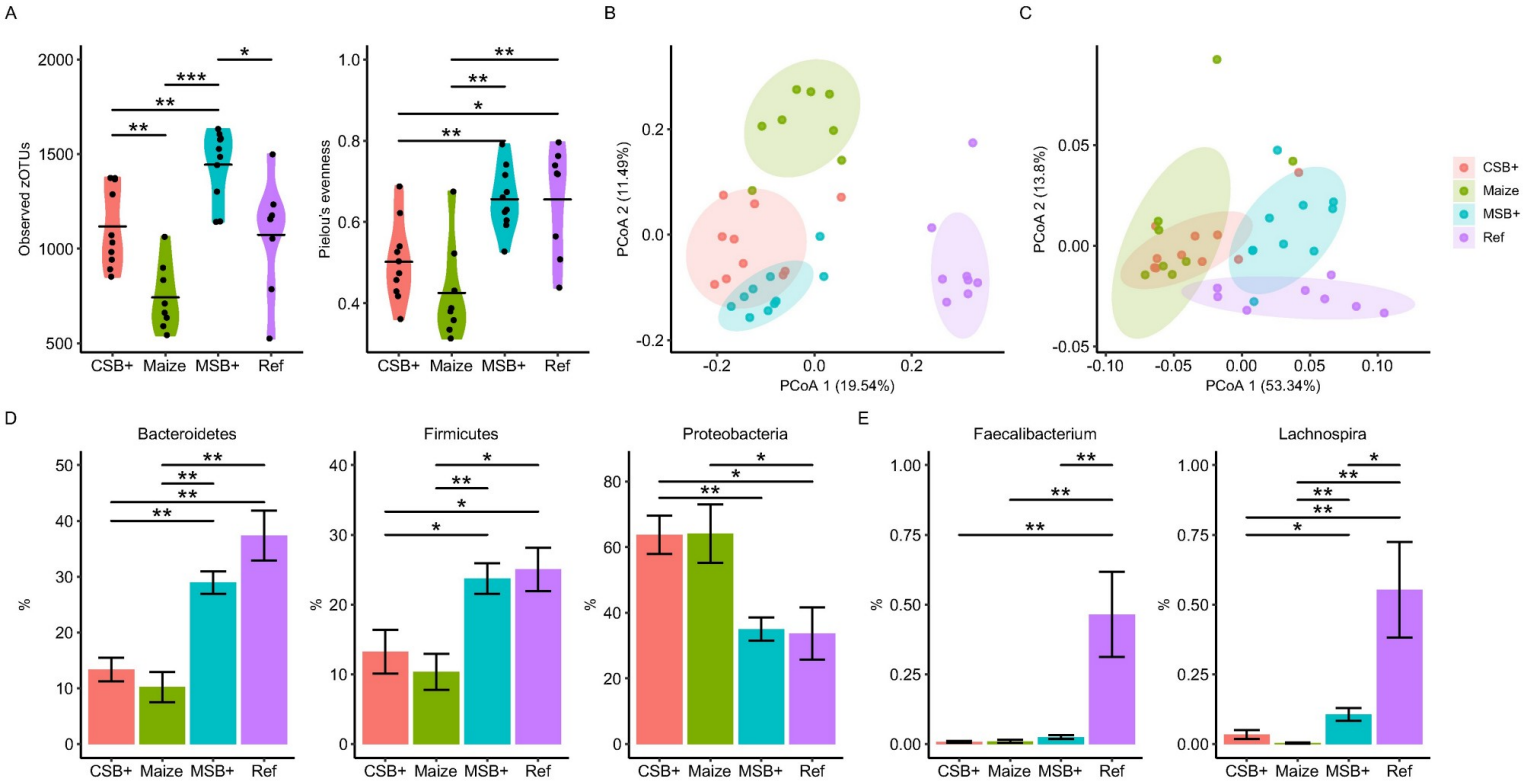
Colon mucosal microbiota shifts determined by the 16S rRNA gene amplicon sequencing. Observed zOTU numbers and Pielou’s evenness (A). PCoA plot of unweighted (B) and weighted (C) UniFrac distance metrics; the colored ellipses indicate 80% confidence level following multivariate t-distribution. The relative abundance of *Bacteroidetes*, *Firmicutes* and *Proteobacteria* between groups (D). Differentially abundant *Faecalibacterium* and *Lachnospira* spp. identified by ANCOM. (E). For CSB+ and MSB+, *n* = 10; For Maize and Ref, *n* = 8. Data in the bar plot was shown by mean value together with SEM error bar. The labels of *, **, *** represent adjusted p < 0.05, < 0.01 and < 0.005 respectively.

The colon mucosal microbial structure was profiled by 16S rRNA gene amplicon sequencing. Total observed zOTU number and Pielou’s evenness were calculated to evaluate the alpha diversity (Fig 3A). Compared with Ref, Maize pigs harbored a microbial community of lower richness and biased composition (*p* = 0.05 and < 0.05, respectively). CSB+ and MSB+ refeeding increased the microbiota diversity of malnourished pigs (*p* < 0.05, 0.005, respectively) to a level comparable or even higher than the Ref pigs. Unweighted and weighted UniFrac distance metrics indicated distinct difference between malnourished pigs and Ref (Fig 3B and 3C). We conducted pairwise PERMANOVA tests on unweighted UniFrac distance metrics and found all 4 groups were significantly different to each other (Adjusted *p* < 0.001). And CSB+ and Maize were more similar when giving weights according to the bacterial abundance (Adjusted *p* > 0.30, Fig 3C). *Firmicutes*, *Bacteroidetes* and *Proteobacteria* were three main phyla found in the pigs, where no significant differences were found between MSB+ and Ref. In contrast to these two groups, CSB+ and Maize (P > 0.05) were much similar with distinct reduction of *Bacteroidetes* and *Firmicutes* but increased *Proteobacteria* abundance (Fig 3D). The relative abundance of *Faecalibacterium* and *Lachnospira* were different under different feeding regimes (Fig 3E). *Faecalibacterium* and *Lachnonspira* remained at a lower level in the malnourished pigs relative to Ref (P < 0.05), nevertheless, MSB+ feeding significantly increased *Lachnospira* abundance relative to CSB+ and Ref.

### Pearson’s correlation analysis between phenotype and mucosal microbiota

**Fig 4:**
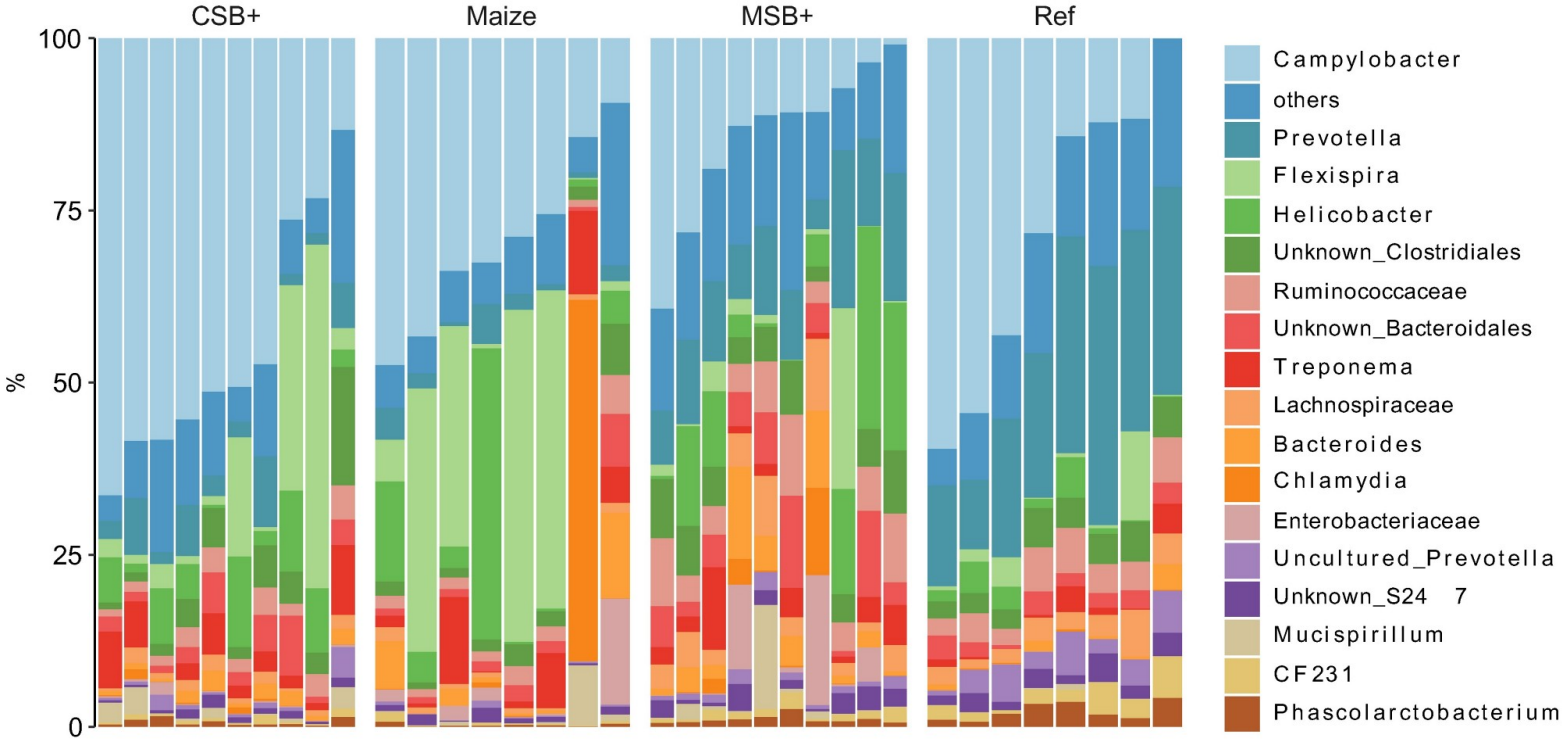
Relative abundance of colon mucosal microbiome members summarized at genus level. For CSB+ and MSB+, *n* = 10; For Maize and Ref, *n* = 8.

**Fig 5:**
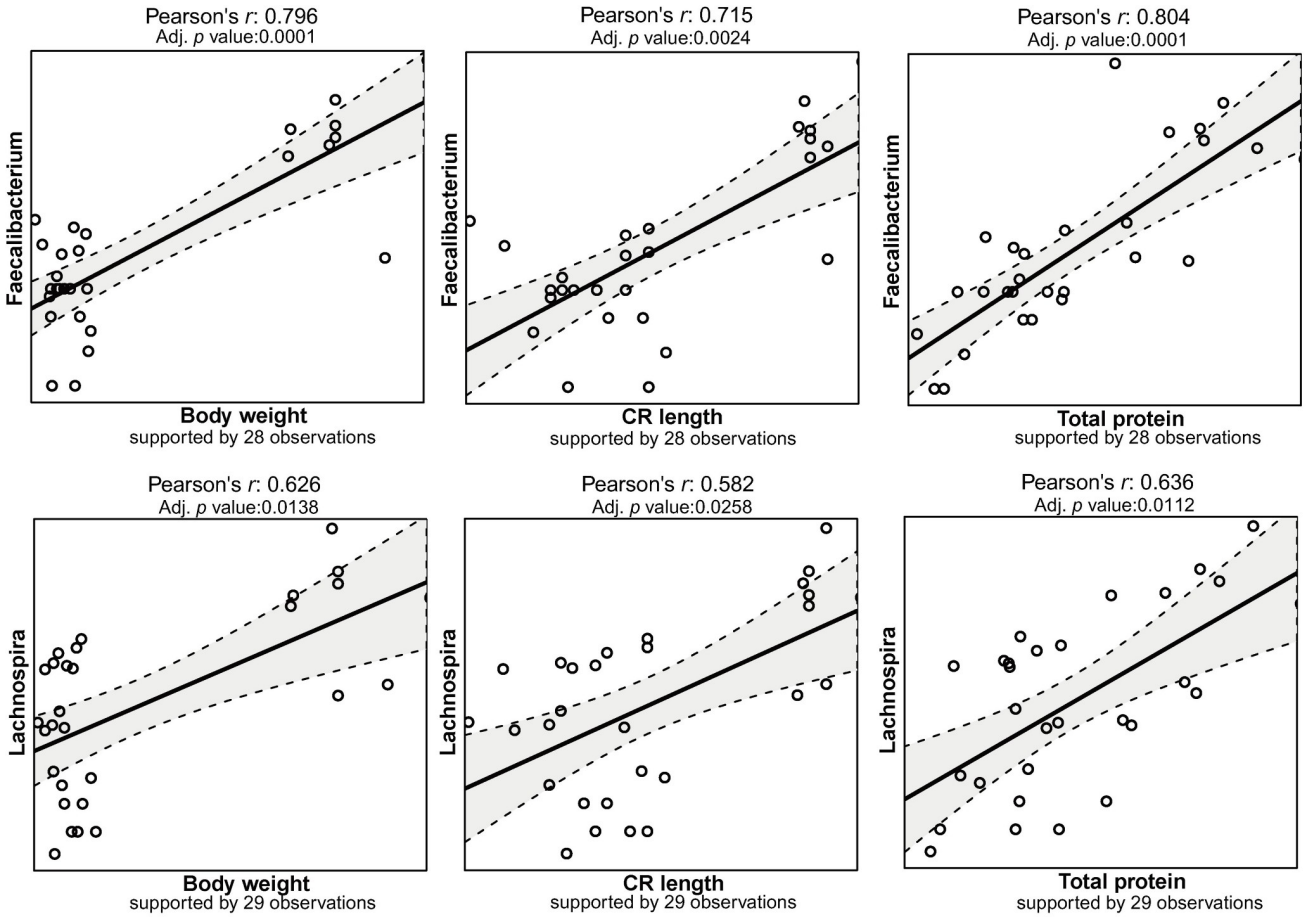
The Pearson’s correlation matrix between host body development and the abundance of *Faecalibacterium* and *Lachnospira*. The observation number indicated the number of samples included in the correlation analysis. Zeros in taxonomic abundance were treated as missing values. Samples with missing values were excluded for the correlation analysis.

The rarefied zOTU table was collapsed at genus level resulting in 103 annotated taxa, of which 58 were unambiguously identified to family and upper levels. *Campylobacter*, *Prevotella*, *Flexispira* and *Helicobacter* occupied the mucosal samples from these pigs, taking up more than half of the microbial community. For CSB+ and Maize, *Campylobacter* and *Flexispira* spp. accounted for the high abundance of *Proteobacteria* while decreased levels of these in MSB+ and Ref were replaced by increased relative abundance of *Helicobacter* and other bacteria e.g., *Prevotella* spp. (Fig 4). A total of 76 correlations between the mucosal microbiome and host traits remained significant after multiple tests adjustment (adjusted *p* < 0.05, S2 Table). In comparison to other phenotypic data, body weight, CRL, total protein, albumin and creatinine were closely related to the microbiome changes (S6 Fig). The abundance of *Lachnospira*, *Faecalibacterium* showed strong positive correlation to the body weight, CRL, total protein with Pearson’s coefficient > 0.5 (Fig 5).

### Microbial functional capacity profiling with PICRUSt 2

**Fig 6:**
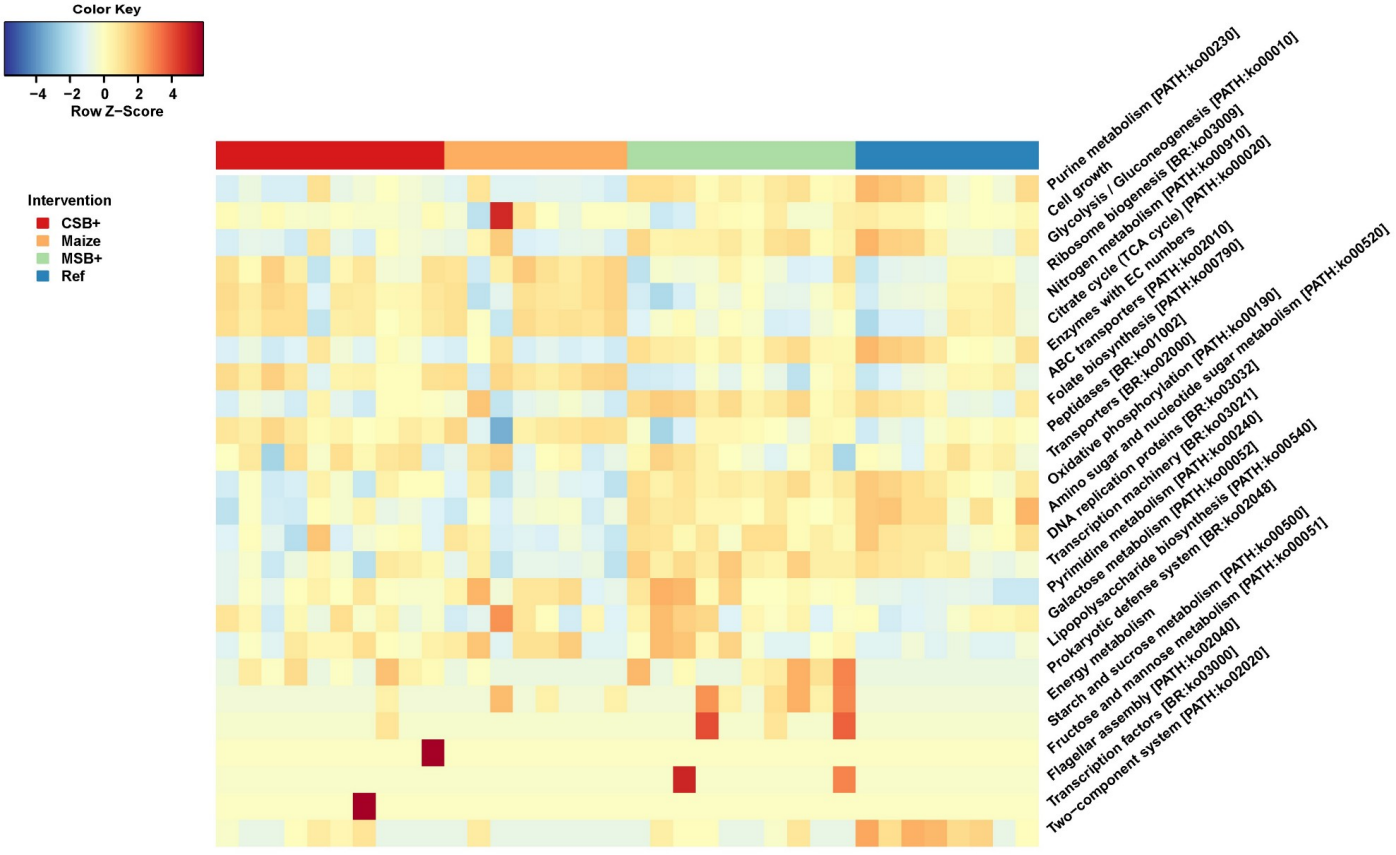
Microbial functional capacity predicted by PICRUSt 2. The heatmap showing the differentially enriched pathways identified by LEfSe (LDA scores > 3). The relative abundances of KEGG pathways were visualized in heatmap after Z-score normalization. For CSB+ and MSB+, *n* = 10; For Maize and Ref, *n* = 8.

PICRUSt 2 was used to predict function KOs based on zOTU sequences and 6332 KOs were predicted with the minimum frequency of 32711943. In order to identify the difference of pathway enrichment, all the KOs were collapsed to 267 pathways and 24 respective pathways were identified with LDA score > 3. Differentially enriched patterns of microbial functional pathways were visualized in heatmap after Z-score normalization (Fig 6). MSB+ resembled the metabolic enrichment of Ref in contrast to CSB+ and Maize. Purine metabolism, glycolysis/gluconeogenesis, folate biosynthesis, oxidative phosphorylation and other bacterial metabolic pathways were enriched in both MSB+ and Ref. Reversely, nitrogen metabolism, citrate cycle, and ABC transporters were mainly enriched in CSB+ and Maize but not in MSB+ and Maize.

## Discussion

### Three-week refeeding of MSB+ and CSB+ showed limited phenotypic improvements in the malnourished pigs

Children are the most vulnerable group to inadequate nutrition due to their poorly developed immune system and puberty lateness [35, 36]. Malnourished children usually need to ingest enough calories to sustain body development, while on the opposite, they mostly face serious digestive problems or lack of appetite at the same time [37]. In this study, we used a pig model to mimic the malnutrition symptoms in body development, followed by three-week nutritional supplementation of CSB+ and MSB+. During this period, malnourished pigs showed distinct phenotypic difference incl. stunted growth relative to pigs fed by Ref. Even in the MSB+ and CSB+ group, pigs still manifested most malnourished symptoms e.g., low body and organ weight and CRL, which might be associated with inadequate feed intake of these pigs relative to Ref. CSB+, MSB+ and Maize pigs exhibited comparable level for most phenotypic evaluations incl. biochemical profiles, systemic proinflammatory cytokines and gut morphology. CSB+ and MSB+ pigs only showed elevated blood concentrations of total protein and globulin relative to Maize. These suggested that three-week refeeding only led to mild improvements of malnutrition symptoms, and extended intervention might be needed to see the distinct improvements in physiological change.

### Millet-based supplementation shifted the aberrant colon mucosal microbiota of malnourished pigs

Both CSB+ and MSB+ significantly increased the colon mucosal microbiota richness, and MSB+ restored the species evenness as well. Compared with CSB+, MSB+ had more diverse and evenly-distributed commensal microbiome, i.e., a steadier system. Moreover, MSB+ pigs showed well-fed like microbial composition in colon mucosa with increased *Bacteroidetes* and *Firmicutes* abundance but reduced *Proteobacteria*. Decreased diversity and potential pathobionts from *Proteobacteria* were reported characteristics in malnourished children’s microbiota [38, 39]. Decreased abundance of *Campylobacter* and *Flexispira* corresponded to the less *Proteobacteria* in MSB+ and Ref, which were substituted by *Helicobacter*, *Prevotella* and other bacteria from *Firmicutes* and *Bacteroidetes*. The natural property of millet increased the vitamin B2, B6 and magnesium in MSB+, but decreased levels of vitamin D, vitamin K, biotin and iodonium. The component difference in feed might account for these microbial shifts. Besides those, the level of propionic acid in colon, was found significantly increased in MSB+ pigs relative to Maize. Propionic acid mainly derived from fermentation of dietary fiber by colonic microbes. Several studies have reported its anti-inflammatory properties [40] and the ability to inhibit pathogenetic bacteria colonization [41]. The increased propionic acid level suggested that the altered microbiota by MSB+ refeeding might improve the pathogen resistance capacity of these malnourished pigs.

### The relative abundances of *Faecalibacterium* and *Lachnospira* spp. were positively associated with the malnutrition-associated phenotype

Pearson’s correlation analysis indicated the specific bacterial abundances were associated with the malnutrition-associated phenotype e.g., body weight, CRL, total protein, albumin and creatinine. *Lactobacillus*, *Lachnospira*, *Faecalibacterium* and *Roseburia* spp. relative abundance were positively correlated with these indexes, suggesting the lower the abundance of these taxa, the more serious the malnutrition status was. Previously, children with low levels of *Lachnospira*, *Veillonella*, *Faecalibacterium* and *Rothia* in their first 3 months of life have been found to have higher risk of asthma [42]. Similarly, *Faecalibacterium prausnitzii* has been found in low abundance in the gut of malnourished children in Bangladesh as well as the malnourished pigs [43]. Promisingly, MSB+ significantly increased gut abundance of *Lachnospira* spp. relative to CSB+ and Maize, which has been found to be associated with the anti-inflammatory effect in weaner pigs fed by a high-fiber diet [44].

### MSB+ pigs shared similar functional gene enrichment in microbial community relative to Ref

Along with shifted microbial structure, MSB+ pigs showed similar microbial metabolic patterns relative to Ref. The GM of MSB+ and Ref pigs showed distinct genetic enrichment in purine metabolism, glycolysis/gluconeogenesis, oxidative phosphorylation and folate biosynthesis. The improved microbial diversity shall take account for the enriched central energy metabolism pathways e.g., purine metabolism, glycolysis/gluconeogenesis and oxidative phosphorylation, suggesting a more active and cooperative bacterial food web [45, 46]. Meanwhile, the food intake allows commensal microbes to complete necessary metabolism and biosynthesis, which nurtures the host. Evidence is growing, that commensal microbes also contributes e.g., folate biosynthesis, especially at the early stage of children maturation [47]. Unlike MSB+ and Ref, nitrogen metabolism, citrate cycle, and ABC transporters were the main pathways with increased abundance in CSB+ and Maize, suggesting the metabolic direction of microbial community differed upon the source of intake.

## Conclusion

Malnourished pigs manifested stunted development and aberrant colon mucosal microbiota relative to pigs fed by standard formulated rations. After three-week refeeding, both corn-soy blend and millet feed improved the concentrations of total protein and globulin in blood but these pigs still manifested most malnutrition symptoms. And millet showed the promising benefits as material source in modulating the GM dysbiosis of the malnourished pigs.

## Supporting information

S1 Fig Average feed consumption by pen. These pigs had *ad libitum* access to the feed and the feed were added and recorded by pen during the experiment. The feed consumption per pig was estimated by the accumulated feed consumption divided by the number of pigs in respective pens.
(PDF)

S2 Fig Malnutrition index relative to Ref. For CSB+ and MSB+, *n* = 10; For Maize, *n* = 8. Data in the bar plot was shown by the mean value together with SEM error bar.
(PDF)

S3 Fig Histopathological assessment criteria of distal colon samples. (PDF)

S4 Fig Colon mucus layer thickness. For CSB+ and MSB+, *n* = 10; For Maize and Ref, *n* = 8. Data in the bar plot was shown by the mean value together with SEM error bar. The labels of * represents adjusted *p* < 0.05 respectively.
(PDF)

S5 Fig Distal colon intestine cell profiling by AB-PAS staining. For CSB+ and MSB+, *n* = 10; For Maize and Ref, *n* = 8. Data in the bar plot was shown by the mean value together with SEM error bar. The labels of * represents adjusted *p* < 0.05 respectively.
(PDF)

S6 Fig Pearson’s correlation between phenotypic data and genus-level bacterial composition in colon mucosa. Centered log ratio transformation was applied before calculating the Pearson’s correlation. In the heatmap, the dot size and color depth indicate the unadjusted *p* value and Pearson’s coefficient, respectively.
(PDF)

S1 Table The detailed pairwise comparisons of phenotypic data between treatment groups. (XLSX)

S2 Table The significant correlation pairs between the colon mucosal microbiome and host traits after multiple tests adjustment. (XLSX)

## Abbreviations

ANCOM: Analysis of compositions of microbiomes
CRL: Crown-rump length
CSB+: Corn soy blend with extra micronutrients
GM: Gut microbiota
KEGG: Kyoto encyclopedia of genes and genomes
KO: KEGG Orthology
LDA: linear discriminant analysis
LEfSe: Linear discriminant analysis effect size
MSB+: Millet soy blend with extra micronutrients
PCoA: Principal coordinate analysis
PERMANOVA: Permutational multivariate analysis of variance
PICRUSt: Phylogenetic investigation of communities by reconstruction of unobserved states
QIIME: Quantitative insights into microbial ecology
RUSF: Ready-to-use supplementary food
SCFA: Short chain fat acid
SD: Standard deviation
SEM: Standard error of the mean
zOTU: Zedius-zero operational taxonomic unit
