# Supplementary figures and images for: Millet-based supplement restored gut microbial diversity of acute malnourished pigs
Source: PLoS One. 2021 Apr 29;16(4):e0250423. doi: 10.1371/journal.pone.0250423 (PMC8084169; doi:10.1371/journal.pone.0250423)

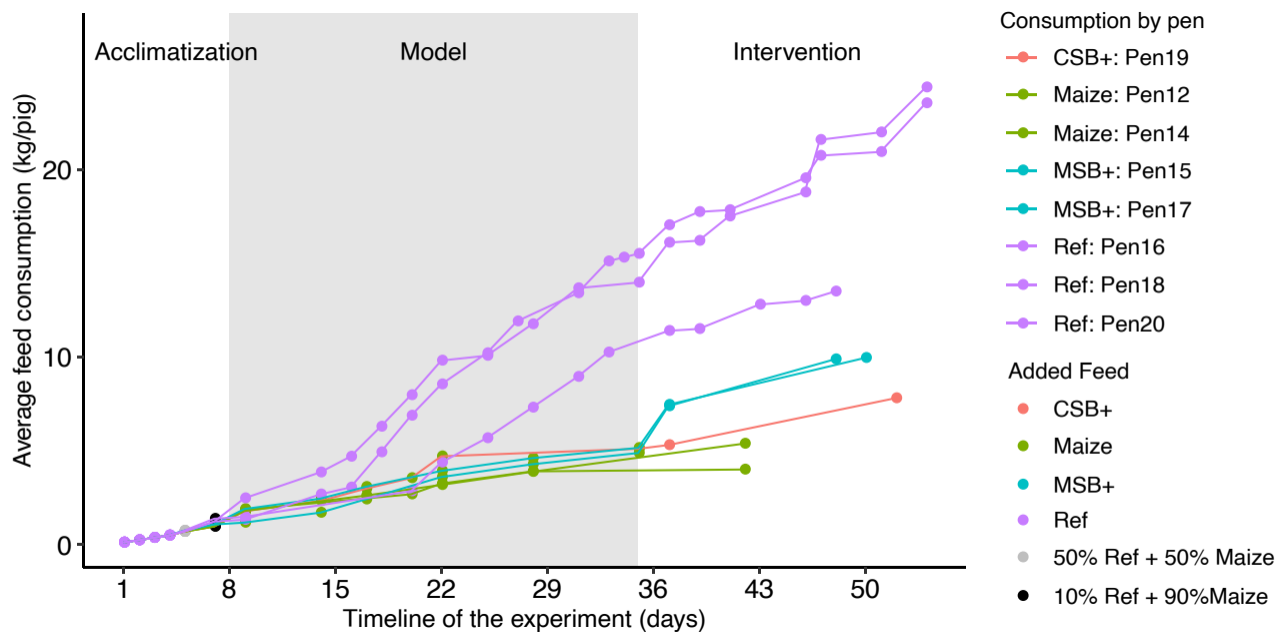

Supplement: S1 Fig — These pigs had ad libitum access to the feed and the feed were added and recorded by pen during the experiment. The feed consumption per pig was estimated by the accumulated feed consumption divided by the number of pigs in respective pens. (PDF) [file pone.0250423.s001.pdf]

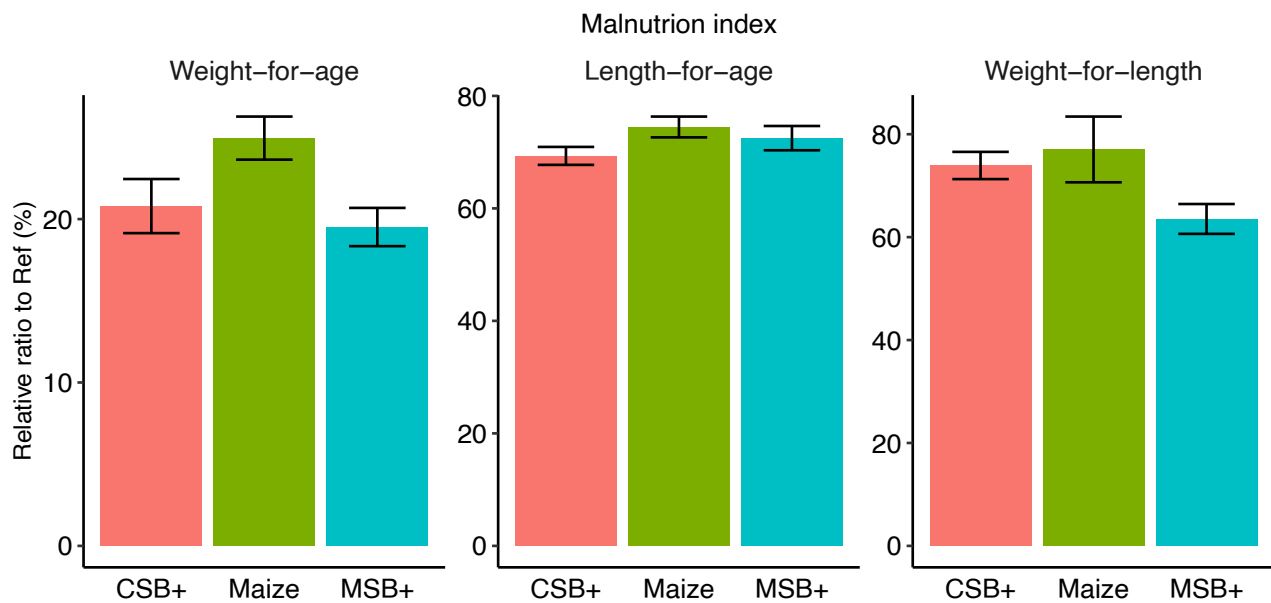

Supplement: S2 Fig — For CSB+ and MSB+, n = 10; For Maize, n = 8. Data in the bar plot was shown by the mean value together with SEM error bar. (PDF) [file pone.0250423.s002.pdf]

# Colon mucus layer thickness

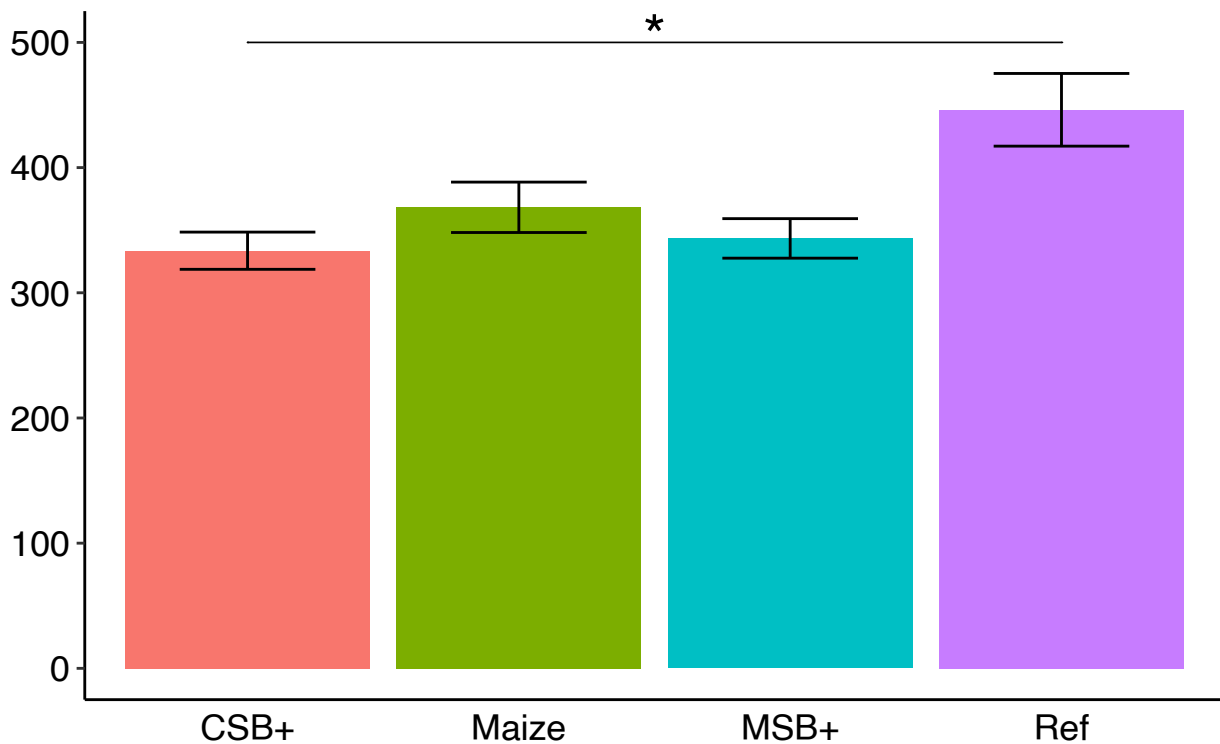

Supplement: S4 Fig — For CSB+ and MSB+, n = 10; For Maize and Ref, n = 8. Data in the bar plot was shown by the mean value together with SEM error bar. The labels of * represents adjusted p < 0.05 respectively. (PDF) [file pone.0250423.s004.pdf]

# Distal Intestine Cell Profiling by AB-PAS

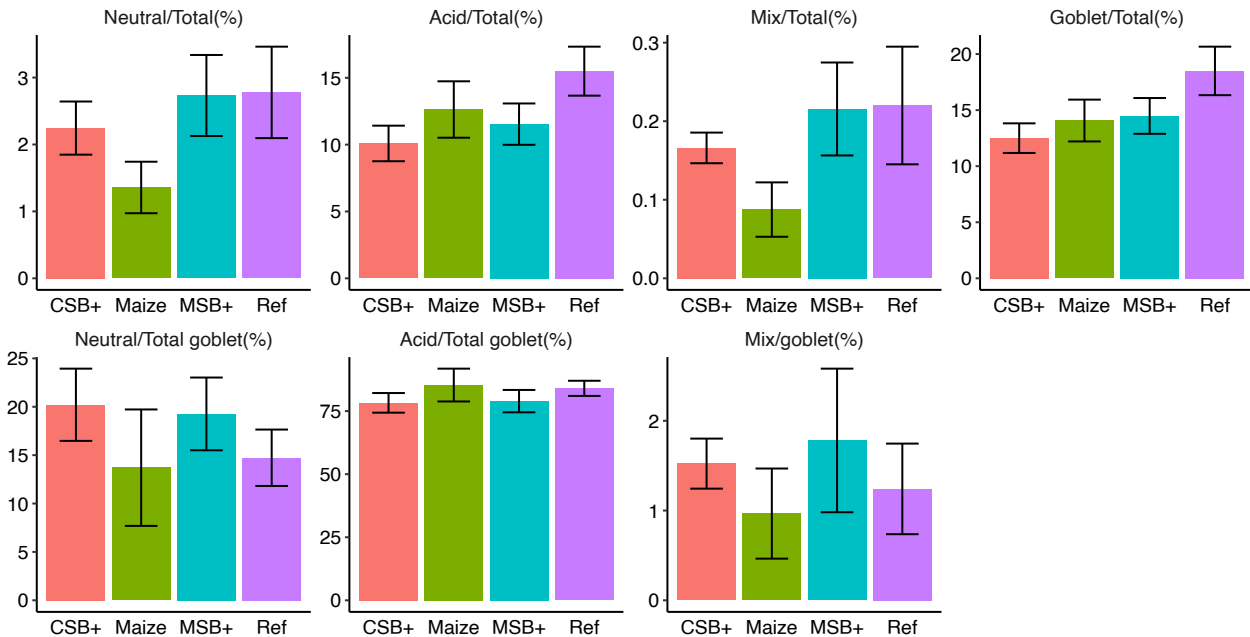

Supplement: S5 Fig — For CSB+ and MSB+, n = 10; For Maize and Ref, n = 8. Data in the bar plot was shown by the mean value together with SEM error bar. The labels of * represents adjusted p < 0.05 respectively. (PDF) [file pone.0250423.s005.pdf]

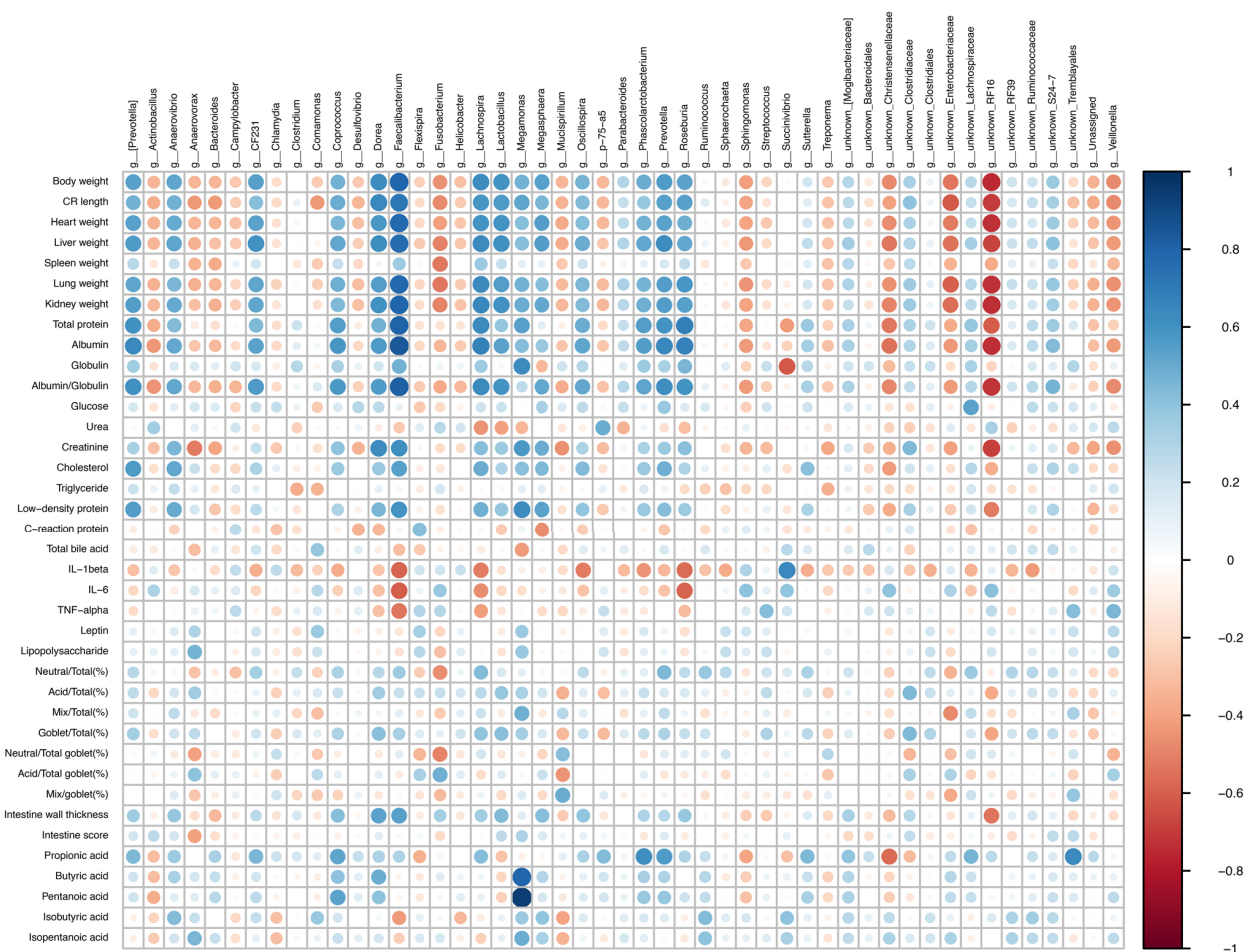

Supplement: S6 Fig — Centered log ratio transformation was applied before calculating the Pearson’s correlation. In the heatmap, the dot size and color depth indicate the unadjusted p value and Pearson’s coefficient, respectively. (PDF) [file pone.0250423.s006.pdf]
