# Supplementary material for: Millet-based supplement restored gut microbial diversity of acute malnourished pigs
Source: PLoS One. 2021 Apr 29;16(4):e0250423. doi: 10.1371/journal.pone.0250423 (PMC8084169; doi:10.1371/journal.pone.0250423)
Supplement: S3 Fig — (PDF) [file pone.0250423.s003.pdf]

Normal	The surface epithelial cells, mucosa and submucosa of the colon were normal.	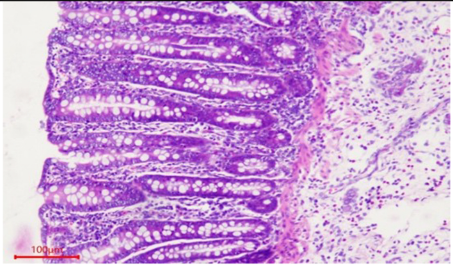
Mild	The thinned epithelial layer is small focus, and a small area of the mucosal layer is infiltrated with red serous fluid in the glandular cavity and surrounding interstitial. Occasionally, the lymphatics is dilated with some thin pink serous fluid.	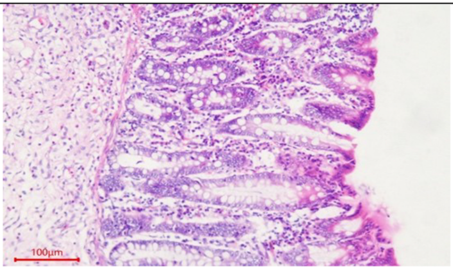
Moderate	The multifocal histological lesions showed as thinned epithelial layer with partly bacterial adhesion, resulted from the necrosis and defulvium of mucosal epithelia. Multifocal serous were infiltrating in the mucosal layer, and the dilated lymphatics filled with pink serous fluid in the submucosa.	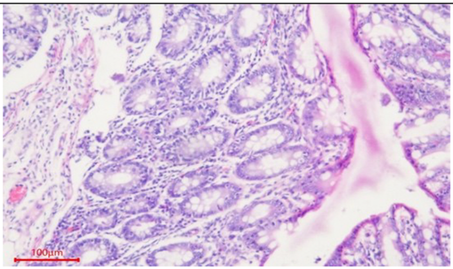
Severe	The multifocal histological lesions appeared as thinned epithelial layer with bacterial adhesion, resulted from the necrosis and defulvium of mucosal epithelia. There was deep-red serous fluid infiltrating in the mucosal layer, and some significantly dilated lymphatics could be seen in the interstitial of intestinal gland and submucosa.	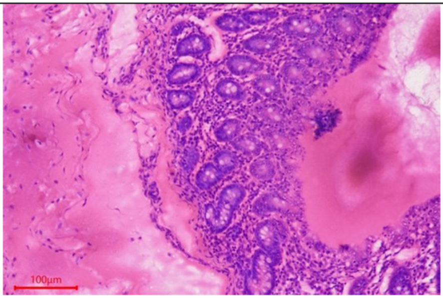
